# Comment on “Efficacy of a Large Language Model Data Extraction System in Evidence Reviews for Emerging Infectious Diseases: A Randomized Crossover Trial”

**DOI:** 10.1093/ofid/ofag653

**Published:** 2026-10-01

**Authors:** Douglas W Challener, John C O’Horo

**Affiliations:** Division of Public Health, Infectious Diseases, and Occupational Medicine, Mayo Clinic Rochester, Minneapolis, Minnesota, USA; Division of Public Health, Infectious Diseases, and Occupational Medicine, Mayo Clinic Rochester, Minneapolis, Minnesota, USA

To the  Editor—Ishikane et al evaluated the important task of using a large language model (LLM)–based tool in the context of data extraction for consolidation of evidence for infectious disease–related systematic reviews [1]. We commend the authors for the study idea and the transparency of releasing their code on Github. Unfortunately, we believe this study fell short in a number of ways that we would like to highlight.

The authors used OpenAI's o3 model to process the first 10 000 characters of article PDF text into 7 fields: filename, theme, category, time, place, person, and a brief bullet summary. Notably, all of these fields could easily be answered from the papers’ abstract and none are explicitly returning quantitative data (no estimate of effect, denominators, outcome definitions, etc). The statement by the authors about data extraction being perfect (100%) is more about capture of bibliographic information than data.

The data extracted from the article PDFs were limited to the first 10 000 characters. The problem with this approach is that this disadvantages the LLM tool. The 10 000 character cap would include the PDF's front matter, abstract, etc, but often would miss the later parts of the manuscript. We reconstructed the pipeline published by the authors. Of the 4 articles [2–5], 2 were narrative reviews without any results sections [2, 3]. Neither of the 2 original research articles had their results section fully captured by the character truncation (Table 1). The results section of the Ishikane study mentions a total of 6 papers, but the data made available for review only have 4 paper identifiers.

**Table 1.** Articles Evaluated by Ishikane et al

Paper	Article Type	Total Characters in PDF	Retained by 10 000 Character Limit	Results Section Captured?
Tecovirimat resistance [4]	Original research	33 459	29.9%	Partially—the results section runs from character 7689 through 13 107
Monkeypox [2]	Narrative review	52 283	19.1%	No results section
JYNNEOS effectiveness [5]	Original research	36 322	27.5%	No—the cut lands in the header of Table 1
Mpox respiratory transmission [3]	Narrative review	45 953	21.8%	No results section

The authors’ accuracy endpoint (stated at 100% accurate) is also questionable. In the supplied anonymized results, participant 004 reported the enrollment period of the JYNNEOS study as 19 April 2022 through 31 March 2023. The correct enrollment period (available in the title of the study) began in 19 August of that year. The same reviewer reported 309 vaccinated patients, whereas the 309 patients actually represented the case group in the case-control study. This reviewer's summary was scored as accurate. Interestingly, the results also show that 1 participant (P003) was reassigned to an article that he had previously summarized 11 days earlier (first time was 58 minutes without AI assistance, then 36 minutes with assistance). That paired assessment contributes nearly 3 times the overall reported effect in reduced time, suggesting that this reassignment benefited from the prior effort.

There is also a confounding issue of translation. Japanese reviewers in the intervention arm were reading a Japanese-language summary of the articles, though the control arm was reading English full text. The authors mention that the non-LLM group could use translation software “but the use of artificial intelligence (AI) tools was not (permitted)”. Modern machine translation tools use neural network techniques, so this is a distinction without a difference.

Despite these limitations, this does not reduce the importance of new LLM-based workflows in data extraction and evidence synthesis. Ideally, the study would be performed with a schema including quantitative fields for extraction, full text evaluation of original research studies using a modern frontier LLM, language held consistent across arms, and independent adjudication of the extraction against the source. Extraction benchmarks should be judged by performance in the results section of manuscripts as this is the section used for data synthesis. If a cap of characters required truncation, a systematic approach limiting to appropriate sections (eg, using only for the abstract review stage and confining summarization/extraction to abstract elements) would be much more useful.

The trial supports the feasibility of a pregenerated summary as part of a pipeline for data synthesis reviews. However, it does not establish that LLM extraction preserves accuracy on the very most important data elements. The study is worth repeating on a larger scale with these issues addressed.
